# Measurement of gender inequality in neighbourhoods of Québec, Canada

**DOI:** 10.1186/1475-9276-10-52

**Published:** 2011-11-16

**Authors:** Lum Tamambang, Nathalie Auger, Ernest Lo, Marie-France Raynault

**Affiliations:** 1 Department of Social and Preventive Medicine, University of Montréal, 1420 boulevard Mont-Royal, Montréal, Québec H2V 4P3, Canada; 2 Institut national de santé publique du Québec, 190 boulevard Crémazie Est, Montréal, Québec H2P 1E2, Canada; 3 Research Centre of the University of Montréal Hospital Centre, 3850 Rue Saint-Urbain, Montréal, Québec H2W 1T7, Canada; 4 Direction Prévention et Santé Publique de l'Agence de la santé et des services sociaux de Montréal, 1301 Sherbrooke Est, Montréal, Québec H2C 1M3, Canada

**Keywords:** Aggregate data, Gender inequality, Population surveillance, Residence characteristics, Rurality

## Abstract

**Introduction:**

Few studies have measured gender inequality at levels lower than the country. We sought to develop neighbourhood indicators of gender inequality, and assess their ability to capture variability in gender inequality across Québec, Canada.

**Methods:**

Aggregate 2001 census data for 11,564 neighbourhoods were obtained for men and women. Twelve indicators of gender inequality representing demographic/household characteristics, education, income, work/leisure, and political participation were selected. Neighbourhood-level gender inequality scores were computed for each indicator, and examined across parts of Québec (metropolitan areas, mid-sized cities, rural areas). Monte Carlo simulations were used to assess the ability of indicators to capture heterogeneity in gender inequality across neighbourhoods.

**Results:**

Male-dominant neighbourhood-level gender inequality tended to be present for average employment income, labour force participation, employment rate, and employment in managerial positions. Female-dominant gender inequality tended to be present for divorce, single-headed households, and participation in unpaid housework, child and elderly care. Neighbourhood-level gender inequality tended to vary across metropolitan areas, mid-sized cities, and rural areas. Gender inequality scores also varied within these geographic areas. For example, there was greater income-related gender inequality in high than low income neighbourhoods. Monte Carlo simulations suggested that the variation in gender inequality across neighbourhoods was greater than expected with chance alone.

**Conclusion:**

Neighbourhood-level gender inequality tended to be present in Québec, and varied across parts of the province. Greater awareness of and research on neighbourhood-level gender inequality may be warranted to inform gender policies in Québec and other nations.

## Introduction

Achieving gender equality is a leading millennium development health goal [1,2]. In Canada, efforts to promote gender equality in education and employment have been numerous [3], but few studies have investigated the influence of such policies in populations. Canada ranked sixteenth worldwide on the United Nations' gender inequality index in 2008 [4], and several Canadian studies have shown important gender differences in chronic illness, distress, mental health, and self-rated health [5,6]. The potential for gender inequality to be related to these outcomes has not been investigated, despite recognition of gender as a determinant of health [7].

How to measure gender inequality in industrialized countries is an emerging topic in literature [4,8-10]. Gender is a social construct related to roles, relationships, personality traits, attitudes, relative power, and other characteristics ascribed to men and women by society [7]. Gender inequality refers to unequal relationships between men and women with regard to resource distribution, responsibilities and power [11]. Researchers have proposed indicators such as the gender inequality index and global gender gap index [4,8], but lack of data in many countries limits applicability. In addition, these indicators are measured at the country level [4,8,10,12], and cannot be used to capture gender inequality at lower levels. Studies have documented that neighbourhood-level characteristics (e.g., income, poverty, education) may be associated with health [13-18], and even that neighbourhood levels of female education may be more strongly associated with adverse birth outcomes than are male levels [13]. US and Swedish studies found that state- and municipal-level gender inequality measured with aggregated census data were associated with low birth weight, infant mortality, depression, and adult mortality [19-22], but indicators for smaller area levels (or neighbourhoods) have not been considered. Developing indicators to assess gender inequality at lower levels would be particularly relevant in Canada where neighbourhood-level data are available from the census. Such indicators may also enable urban-rural comparisons. Health disparities vary across neighbourhood characteristics of urban and rural areas [23-29], but the potential contribution of gender inequality has not been investigated. Assessing actual levels of neighbourhood gender inequality is important for creating awareness of its potential presence, and to improve gender policies in Québec. Such analyses may also stimulate development of alternate measures of gender inequality for surveillance or future research investigating the causal pathways linking neighbourhoods and health.

Given lack of data on how gender inequality may vary across neighbourhoods of Canada, we sought to develop neighbourhood indicators to determine if gender inequality is present in the province of Québec, and assess their ability to capture variability in gender inequality across parts of Québec. We selected indicators based on the general feminist theory which emphasizes equal participation of men and women in all spheres of life [11]. According to this theory, gender relations between men and women should reflect equal distribution of power and influence in private and public roles, opportunities for financial independence, responsibility for the home and children, access to education and development of personal interests without coercion or intimidation. This approach translates into five dimensions of gender inequality related to 1) demographic and household characteristics, 2) education, 3) income, 4) work and leisure, and 5) political participation [30]. Since Québec has adopted several policies to promote gender equality in education, income and employment [31], gender inequality may be lower in these domains.

## Methods

### Data

Aggregate data available for 11,612 Québec dissemination areas (DA) were extracted from the 2001 population census produced by Statistics Canada. Census data were collected from 100% of households for demographic indicators, and 20% of households for remaining indicators with data weighted to be representative of the entire population. DAs were chosen as proxies for neighbourhoods since they cover all of Québec, and are often used in research as they successfully capture inequalities [32]. DAs contain 400-700 residents on average, and span a maximum of 99 blocks [33]. Twelve DAs missing data were excluded, as well as 36 with an exclusively male or female population, for which it was impossible to compute gender inequality scores. The final sample consisted of 11,564 DAs. A subset of these (N = 15) with no population in the labour force were excluded from analyses involving labour force indicators. Census data were examined for three area types (metropolitan, mid-sized cities, rural areas). Metropolitan areas have a population of ≥ 100,000 with ≥ 50,000 in urban cores, whereas mid-sized cities have smaller urban cores with ≥ 10,000 individuals. Rural areas consist of the remainder of Québec, and include small towns with < 10,000 inhabitants [33].

### Variables

For each dimension of gender inequality (demographic and household characteristics, education, income, work and leisure, political participation), we selected variables available in 2001 census data. In total, 12 variables representing the five dimensions of gender inequality were selected (Table 1), and are discussed below.

**Table 1:** Dimensions and definitions of census variables for gender inequality

Dimension	Definition*
**Demographic and household** **characteristics**	
Divorce	Proportion of people aged ≥ 20 years who are divorced
Single-headed households	Proportion of households that are single-headed
**Education**	
University certification	Proportion of people aged ≥ 20 years who are university-certified
No high school diploma	Proportion of people aged ≥ 20 years with no high school diploma
**Income**	
Average employment income	Average income per capita (CAD) from paid employment in year 2000
**Work and leisure**	
Labour force participation	Proportion of people aged ≥ 15 years in the labour force
Employment rate	Proportion of people aged ≥ 15 years in the labour force who are employed
Managerial positions	Proportion of people aged ≥ 15 years employed in managerial positions
Unpaid elderly care	Proportion of people aged ≥ 25 years doing > 5 hours unpaid elderly care per week
Unpaid child care	Proportion of people aged ≥ 25 years doing > 5 hours unpaid child care per week
Unpaid housework	Proportion of people aged ≥ 25 years doing > 5 hours unpaid housework per week
**Political participation**	
Decision-making positions	Proportion of people aged ≥ 15 years in decision-making government positions (judges, lawyers, policy officers)

*Proportions were calculated for men and women separately.

#### Demographic and household characteristics

Knowledge of inequality in demographic and household characteristics is relevant for health policy since such aspects of population structure must be accounted for in health planning [34]. Marital status is associated with health (e.g., mental disorders) [35], and lone parents (especially women) have poorer health compared with two-parent families [36]. Two variables were chosen for this dimension, including the proportion of 1) divorced women versus men aged ≥ 20 years, and 2) female versus male single-headed households.

#### Education

Education enables men and women to achieve autonomy and participate in economic and social development [37]. Two variables capturing opposite ends of the education spectrum were used: the proportion of women versus men aged ≥ 20 years with 1) a university degree (at least a Bachelor's), and 2) no high school diploma.

#### Income

Access to income implies having enough resources to afford food, housing, education and health care [38]. This dimension was measured using average income per capita from paid employment for year 2000 in Canadian dollars (CAD).

#### Work and leisure

Participation in the labour force leads to autonomy and empowerment by increasing access to and control of monetary and non-monetary resources essential for health [38]. Labour market activity (employment in any paid full- or part-time work for the population aged ≥ 15 years, excluding institutional residents, in the week containing the 15^th ^day of the month preceding the census) [39], was measured with two variables: proportion of 1) women versus men in the labour force, and 2) female versus male employment rate. Because of potential occupational sex segregation due to culturally constructed gender roles that influence participation of women and men in professional sectors (e.g., management) [40], we also examined the proportion of women versus men employed in managerial positions.

Moreover, equal division of responsibilities at home may allow women and men to pursue personal and recreational interests. Because unpaid work may prevent participation in such activities [37], we included the proportion of women versus men aged ≥ 25 years doing unpaid 1) elderly care, 2) child care, and 3) housework for > 5 hours per week.

#### Political participation

Gender inequality is reflected in power and control in the political arena [41]. The proportion of women versus men in decision-making positions within the government (judges, lawyers, policy officers) was used for this dimension.

### Gender inequality score calculation

Gender inequality scores for each variable were computed with the formula [female proportion/(female proportion + male proportion)] based on the method proposed by Backhans et al [19]. A score of 0.5 represents perfect gender equality, < 0.5 male-dominant gender inequality, and > 0.5 female-dominant gender inequality. Progressively greater deviations from 0.5 indicate higher levels of inequality. DAs in which proportions were zero for both men and women were attributed a score of 0.5 (since by definition, there were no gender differences). The number of DAs for which this occurred ranged from zero (unpaid elderly care) to 6,752 (decision-making government positions).

### Statistical analysis

For each variable, proportions were computed for women, men and both genders combined (i.e., the overall proportion). Descriptive statistics (mean, standard deviation, median, interquartile range) were computed for proportions and gender inequality scores. The ability of gender inequality indicators to capture variability across neighbourhoods was evaluated in three ways. First, we assessed if the observed distribution in the variance of gender inequality scores was greater than expected under a Monte Carlo simulated null scenario [42], in which gender inequality scores were homogenous across DAs. Gender inequality scores under the null scenario were simulated assuming that count data from DAs were binomially (for proportion-based variables) or normally (for income) distributed. P-values for heterogeneity over and above the variability produced by random differences across DAs were computed. Greater detail on the methodology of Monte Carlo simulations is provided (Additional file 1).

Second, between-area variation was captured using mean gender inequality scores computed by area type (metropolitan, mid-sized cities, rural), and differences were examined with the non-parametric Kruskal Wallis test (since not all census variables were normally distributed). In cases where there were statistically significant differences, the Mann Whitney U/Wilcoxon Ranked Sum test with the Bonferroni adjustment was used to make pair-by-pair comparisons.

Third, within-area variation in gender inequality was examined. We computed Spearman correlation coefficients between each gender inequality score and the corresponding overall proportion (or income level) for the population in the DA. For example, we correlated the overall proportion of people in the labour force with the labour force gender inequality score. This was done to determine whether gender inequality scores were associated with the corresponding overall level of labour force participation, university certification, employment rate, etc in DAs. For indicators that were most correlated (r ≥ 0.4), we stratified DAs into population-weighted tertiles (low, intermediate, high overall proportion), and computed gender inequality scores for each tertile in metropolitan areas, mid-sized cities and rural areas. Statistically significant differences between tertiles were assessed with the Kruskal-Wallis test.

Data analyses were performed with SAS 9.2 and SPSS 17.0. Monte Carlo simulations were performed using R 2.11.1. Data were denominalized, and the requirement for formal ethics approval was waived by the institutional review board of the University of Montréal Hospital Centre.

## Results

Gender inequality scores ranged from 0.0 to 1.0, except scores for average income which ranged from 0.1 to 0.7 (results not shown). Male-dominant gender inequality tended to be present for average employment income, labour force participation, employment rate and employment in managerial positions (Table 2). Female-dominant gender inequality tended to be present for divorce, single-headed households, and participation in unpaid housework, elderly and child care. Gender inequality scores for university certification and no high school diploma had an overall mean of 0.49 and 0.51, respectively, however interquartile ranges were wide. All indicators except divorce and unpaid child care had a variance greater than random (P < 0.01) under the null (homogenous inequality) scenario.

**Table 2:** Descriptive statistics for census variables and gender inequality indicators

	**Female %**		**Male %**		**Gender inequality score**			
	**Mean (SD)**	**Median**	**Mean (SD)**	**Median**	**Mean (SD)**	**Median**	**Interquartile range**	P-value*
	
**Demographic and household** **Characteristics**								
Divorce	11.2 (4.6)	11.0	10.1 (4.0)	10.0	0.53 (0.11)	0.52	0.47-0.59	1.00
Single-headed households	13.7 (9.8)	12.1	3.4 (1.9)	0.0	0.77 (0.26)	0.81	0.60-1.00	< 0.01
**Education**								
University certification	14.7 (12.1)	11.1	16.5 (14.9)	12.0	0.49 (0.21)	0.49	0.39-0.57	< 0.01
No high school diploma	31.1 (15.8)	30.3	30.8 (16.8)	30.0	0.51 (0.13)	0.50	0.45-0.57	< 0.01
**Income**								
Average employment income (thousand CAD)	22.0 (8.7)	21.6	32.9 (16.3)	31.1	0.42 (0.07)	0.41	0.37-0.45	< 0.01
**Work and leisure**								
Labour force participation	57.0 (14.3)	57.3	69.8 (12.7)	70.6	0.45 (0.06)	0.45	0.42-0.48	< 0.01
Employment rate	53.3 (14.2)	53.6	64.3 (13.6)	65.3	0.45 (0.06)	0.45	0.42-0.48	< 0.01
Managerial positions	6.5 (7.1)	6.0	11.4 (9.5)	9.8	0.37 (0.28)	0.39	0.00-0.50	< 0.01
Unpaid elderly care	8.1 (6.3)	7.1	4.5 (4.0)	4.0	0.65 (0.28)	0.61	0.50-1.00	< 0.01
Unpaid child care	34.5 (13.9)	34.0	25.7 (12.4)	25.0	0.58 (0.11)	0.57	0.52-0.63	0.24
Unpaid housework	79.8 (11.1)	81.2	59.1 (13.2)	60.0	0.58 (0.05)	0.57	0.54-0.61	< 0.01
**Political participation**								
Decision-making positions	2.3 (3.9)	0.0	1.7 (3.8)	0.0	0.52 (0.28)	0.50	0.49-0.51	< 0.01

*P-values were Bonferroni-corrected, and values < 0.01 indicate that the distributions of measured gender inequality scores were heterogeneous, over and above the variability produced by random differences across DAs.

Mean gender inequality scores tended to vary across metropolitan areas, mid-sized cities, and rural areas, except for labour force participation, unpaid work and employment in decision-making positions (Table 3). Differences in mean gender inequality scores for divorce and no high school diploma were statistically significant across all three areas (P < 0.001), with female dominance in metropolitan areas (0.55 and 0.52, respectively) compared to male dominance in rural areas (0.46 and 0.48, respectively). A reverse pattern was observed for university certification, with male-dominant gender inequality in metropolitan areas (0.47) but female-dominant gender inequality in rural areas (0.53). For average income, employment rate, and labour force participation, gender inequality was male-dominant and tended to increase (i.e., scores further from 0.5) with movement from metropolitan to rural areas.

**Table 3:** Mean gender inequality scores for metropolitan, mid-sized cities and rural areas

	**Mean (SD)**			**P-value**
		
	**Metropolitan areas**	**Mid-sized cities**	**Rural** **areas**	
	
**Demographic and household** **Characteristics**				
Divorce	0.55 (0.10)	0.51 (0.11)	0.46 (0.11)	< 0.001*
Single-headed households	0.80 (0.26)	0.74 (0.21)	0.69 (0.28)	< 0.001*
**Education**				
University certification	0.47 (0.20)	0.50 (0.20)	0.53 (0.21)	< 0.001*
No high school diploma	0.52 (0.14)	0.49 (0.08)	0.48 (0.09)	< 0.001^†^
**Income**				
Average employment income (thousand CAD)	0.41 (0.06)	0.38 (0.05)	0.38 (0.06)	< 0.001^†^
**Work and leisure**				
Labour force participation	0.45 (0.05)	0.44 (0.05)	0.44 (0.07)	0.09
Employment rate	0.50 (0.05)	0.41 (0.04)	0.40 (0.07)	< 0.001^†^
Managerial positions	0.30 (0.07)	0.32 (0.06)	0.40 (0.03)	< 0.001^‡^
Unpaid elderly care	0.64 (0.29)	0.65 (0.25)	0.65 (0.27)	0.06
Unpaid child care	0.58 (0.11)	0.58 (0.08)	0.59 (0.12)	0.11
Unpaid housework	0.58 (0.06)	0.58 (0.04)	0.58 (0.05)	0.38
**Political participation**				
Decision-making positions	0.53 (0.28)	0.53 (0.29)	0.52 (0.24)	0.07

* Differences between all three areas are statistically significant.

^† ^Mid-sized cities and rural areas are similar but both differ from metropolitan areas.

^‡ ^Metropolitan and mid-sized cities are similar but both differ from rural areas.

Gender inequality scores for average employment income, labour force participation, and unpaid housework were most correlated with the respective overall income level (r = -0.4), labour force participation (r = 0.4), and unpaid housework (r = -0.4) in DAs (Table 4). For all other variables, the correlation with respective gender inequality scores tended to be low (r ≤ 0.27). The gender inequality score for unpaid elderly care was not correlated (r = 0.01) with the overall proportion of unpaid elderly care.

**Table 4:** Correlation between gender inequality scores and the corresponding overall proportion for the population

	**Overall %**		**Spearman correlation**
		
	**Mean (SD)**	**Median**	**r**
	
**Demographic and household characteristics**			
Divorce	10.7 (3.9)	10.5	0.09
Single-headed households	17.1 (10.9)	15.4	0.13
**Education**			
University certification	15.5 (11.5)	13.4	-0.15
No high school diploma	30.9 (15.4)	30.6	-0.10
**Income**			
Average employment income (thousand CAD)	27.8 (11.8)	26.8	-0.40
**Work and leisure**			
Labour force participation	63.2 (12.4)	63.7	0.40
Employment rate	58.6 (12.8)	59.2	0.27
Managerial positions	9.1 (6.9)	8.0	-0.08
Unpaid elderly care	6.3 (4.5)	5.8	0.01*
Unpaid child care	30.1 (12.0)	29.3	-0.13
Unpaid housework	69.7 (10.2)	70.7	-0.40
**Political participation**			
Decision-making positions	1.9 (3.1)	0.0	0.16

*P = 0.2, all other correlation coefficients were statistically significant with P < 0.01

Overall, there tended to be male-dominant gender inequality for average employment income in all income tertiles, but inequality was highest in the high income tertile, especially in rural areas (Table 5). Furthermore, gender inequality differences in average income between metropolitan, mid-sized cities and rural areas tended to be more pronounced in the intermediate income tertile than the high and low income tertiles. Male-dominant gender inequality also tended to be present in all labour force participation tertiles, with greater inequality in the low tertile. Participation in unpaid housework suggested female-dominant gender inequality in all tertiles, but inequality tended to be highest in the low tertile.

**Table 5:** Mean gender inequality scores for average employment income, labour force participation and unpaid housework according to geographic area and tertile

	**Metropolitan areas**	**Mid-sized cities**	**Rural areas**
	
**Average employment income (thousand CAD)**			
Low (6.4-24.3)	0.43	0.42	0.42
Intermediate (24.3-30.3)	0.42	0.39	0.38
High (30.3-263.7)	0.38	0.38	0.37
P-value*	0.008	0.09	0.06
**Labour force participation (%)**			
Low (0.0-58.0)	0.42	0.42	0.42
Intermediate (58.0-69.0)	0.45	0.45	0.44
High (69.0-100.0)	0.47	0.46	0.46
P-value*	0.15	0.17	0.02
**Unpaid housework (%)**			
Low (8.0-66.0)	0.60	0.60	0.61
Intermediate (66.0-75.0)	0.57	0.58	0.59
High (75.0-100.0)	0.55	0.56	0.56
P-value*	0.01	0.25	0.03

* P-value for the comparison between low, intermediate, and high tertiles in each geographic area.

## Discussion

This study is the first, to our knowledge, to identify indicators capturing different dimensions of gender inequality for measuring differentials across neighbourhoods of Québec. Interestingly, neighbourhood-level gender inequality tended to be present despite laws promoting equality of men and women in education, income and employment (e.g., Pay Equity Act, Parental Insurance Act, and Equal Access to Employment Act) [31]. In most neighbourhoods, men tended to dominate women for income, labour force participation, employment rate, and employment in managerial positions. Women tended to dominate men for divorce, single-headed households, and participation in unpaid work. Most indicators suggested differences in gender inequality levels between urban and rural areas of Québec. Generally, neighbourhoods in rural areas and mid-sized cities had greater gender inequality for income and employment rate compared to metropolitan areas, but less inequality for the other indicators.

Few studies have described indicators of gender inequality for local areas. In Sweden, male-dominant municipal-level gender inequality was observed for income and employment in managerial positions [19]. Direct comparisons of levels of gender inequality between Québec and Sweden, however, are not possible since actual scores were not reported (scores were transformed and used in regression models examining various health outcomes). Furthermore, municipalities in Sweden are larger than DAs, and comparisons should be made with caution. The remaining literature has focused on provincial- or country-level gender inequality. Reports have found male-dominant gender inequality in income and employment for provinces of Canada, but decreasing gender inequality in university certification [43,44]. Provincial-level measures of gender inequality may, however, mask patterns in neighbourhoods.

Variations in gender inequality across urban and rural areas, as well as within areas, suggest that mechanisms leading to gender inequality may be multifactorial and complex. Persistent discrimination in rules, practices, and cultural constructs of gender roles over time may be implicated [31,45]. Historically, income and employment have been confined to men, with women assuming domestic duties or less likely to hold managerial positions [46,47]. These patterns may be reflected by relatively fewer women in the labour force, and lower female employment rates [48]. Thus, male-dominant gender inequality in income and employment may not be surprising, and is consistent with previous empirical reports in the US where male-dominant gender inequality was found for pay and employment in supervisory positions [49]. Moreover, greater male-dominant gender inequality in rural areas may be explained by limited job opportunities, with men obtaining the few jobs available [50].

For the demographic dimension, female-dominant gender inequality in single-headed households may be related to changes in social beliefs and practices regarding the family. Studies from the US and Britain have observed increasing trends in lone parenthood related to perceptions of gender roles in the family that have evolved to accept educated and economically independent women who can afford to raise children alone, as well as child custody practices which favour mothers [51,52]. In Québec, female lone parenthood is socially accepted [53], which may explain why we found female-dominant gender inequality in single-headed households. Furthermore, female-dominant gender inequality in divorce may be associated with female emancipation, or less restrictive divorce laws introduced in the 1980s [54]. Inequality was, however, lower in rural areas, possibly because of a stronger influence of Catholic religious traditions which support marriage and parenthood [55,56]. Moreover, stigma attached to divorce in rural areas, or financial constraints, may encourage remarriage.

Female-dominant gender inequality in unpaid housework, child and elderly care was present, but surprisingly did not vary across parts of Québec. This is unexpected because immigrants settle in metropolitan areas, and have a greater tendency for unpaid labour among women [57]. These findings suggest that unpaid labour in women is widespread and not restricted to immigrants alone.

Differences in neighbourhood gender inequality scores may also be related to selection of individuals into neighbourhoods based on individual characteristics (i.e., self-selection) [58]. This may explain the male dominance observed for university certification in metropolitan areas and female dominance in rural areas. Universities are found in metropolitan areas, and women may potentially be more likely than men to return to rural areas after obtaining university certificates. Male-dominant gender inequality for no high school diploma in rural areas may be related to greater high school dropout for men, especially in rural areas where jobs may not require higher education [50].

### Implications for research and policy

Measuring and monitoring variability in gender inequality across areas is important to tailor programs and policies that minimise inequality between men and women. This study is innovative because it provides a set of indicators which can be used to assess different dimensions of neighbourhood-level gender inequality in Canada. Although research is needed to understand the mechanisms behind urban-rural and within-area variations in gender inequality, the indicators developed in this study may help raise awareness of the possibility of neighbourhood-level gender inequality in other developed nations. International comparisons of gender inequality measured at levels lower than country-level are needed. There may also be implications for the neighbourhood literature - the potential for gender inequality to be involved in causal pathways leading to health could be investigated with indicators similar to those identified in this analysis. Reducing gender inequality should be a priority for policy. Future research and policy could consider initiatives that begin by focusing on dimensions where the highest gender inequality was observed in Québec, such as demographic and household characteristics, income, work and leisure.

Limitations of this study include the use of administrative neighbourhoods - the extent to which they represent true neighbourhoods is unknown. Although small in cities, DAs may be large in rural areas where they may be less likely to reflect true neighbourhoods. Future research may consider use of larger urban units to increase urban-rural comparability, such as census tracts. Census tracts, however, are not available for all urban areas [33], and may mask within-area variations in gender-related characteristics [59]. Another important point is that the indicators used in this study are mathematical formulations of gender inequality, and value judgments should be made with caution. For example, the male-dominant gender inequality observed in the employment rate may not necessarily mean that fewer jobs are available to women. Rather, male dominance may reflect greater gender equality if women are entitled to maternal leave, which may artificially reduce the female employment rate. Finally, this study is exploratory. We do not know what constitutes 'true' gender inequality, or what thresholds of inequality would be acceptable in a just society. Studies suggest that gender inequality may be associated with health [19-22], and that there may potentially be a critical point at which gender inequality no longer influences health [19]. Further research is needed to examine the nature of the relationship between gender inequality and health.

## Conclusion

This study found that neighbourhood-level gender inequality tended to be present, and varied across different parts of Québec. In most neighbourhoods, men tended to dominate women for income, employment rate, labour force participation, and employment in managerial positions, whereas women tended to dominate men for divorce, single-headed households, and participation in unpaid work. The indicators identified in this study may be useful for raising awareness on the existence of gender inequality in Canada to improve gender equality policies. Other countries may benefit from examining dimensions that tended to show the highest gender inequality in Québec, including demographic and household characteristics, income, work and leisure, or other dimensions not explored in this study.

## List of abbreviations

CAD: Canadian dollars; DA: Dissemination area; SD: Standard deviation; SAS: Statistical Analysis System; SPSS: Statistical Package for Social Sciences

## Competing interests

The authors declare that they have no competing interests.

## Authors' contributions

LT and NA conceived the study and prepared the data, with contributions from MFR. LT reviewed the literature. EL performed the Monte Carlo simulations. LT analysed the data under guidance from NA and EL. LT and NA wrote, and EL and MFR revised the manuscript for important intellectual content. All authors read and approved the final manuscript.

## Supplementary Material

Additional file 1 **Methodology of Monte Carlo simulations to assess spatial heterogeneity in gender inequality indicators**. This file describes how Monte Carlo simulations were used to assess spatial heterogeneity in gender inequality indicators. Click here for file
