# Supplementary material for: Measurement of gender inequality in neighbourhoods of Québec, Canada
Source: Int J Equity Health. 2011 Nov 16;10:52. doi: 10.1186/1475-9276-10-52 (PMC3239849; doi:10.1186/1475-9276-10-52)
Supplement: Additional file 1 — Methodology of Monte Carlo simulations to assess spatial heterogeneity in gender inequality indicators. This file describes how Monte Carlo simulations were used to assess spatial heterogeneity in gender inequality indicators. [file 1475-9276-10-52-S1.PDF]

Additional file 1. Methodology of Monte Carlo simulations to assess spatial heterogeneity in gender inequality indicators

Variability of gender inequality scores over dissemination areas (DA) will exist due to statistical randomness, even under the null scenario that the actual 'true' values of the scores do not vary. To determine if there is non-random heterogeneity, the measured variability of gender inequality scores must be greater than the variability under the null scenario. To verify this, the following alternative hypothesis (H_1) is tested against the null hypothesis (H_0), where σ_k and σ_{k_null} are the measured and null variances, respectively, of gender inequality scores over all DAs:

$$H_0\text{: } \sigma_k = \sigma_{k\_null}$$

$$H_1\text{: } \sigma_k > \sigma_{k\_null}$$

Since the null distribution of the gender inequality scores is not known, Monte Carlo simulations are used to generate probability distribution functions for σ_{k_null}, from which P-values for testing H_1 vs. H_0 are obtained.

Monte Carlo simulations for proportion-based gender inequality indicators

Under the null scenario, the 'true' value of the gender inequality score k_{null} is the same over all DAs (note however that the proportions for men and women are allowed to vary between DAs). For simulation purposes, k_{null} is estimated from the median of the measured gender inequality scores over all DAs. The counts of men and women used to calculate the gender inequality scores are assumed to be binomially distributed:

$$X_{im} \sim Bin\left(n_{im}, p_{im\_null}\right)$$

$$X_{if} \sim Bin\left(n_{if}, p_{if\_null}\right),$$

where X_{im} and X_{if} are simulated counts of men and women respectively in the ith DA. The parameters n_{im} and n_{if} are the total numbers of men and women in the ith DA taken from the census, while p_{im_null} and p_{if_null} are the binomial proportions under the null scenario for each DA, calculated from the gender inequality score (k_{null}) under the condition that the total counts of men and women equal the observed total counts ($X_{im} + X_{if} = X_{im\_obs} + X_{if\_obs}$).

Counts of men and women are simulated for each DA, and the variance in the resulting gender inequality scores over all DAs is computed. The simulation is repeated 1000 times to generate a distribution of the variance in the gender inequality scores under the null scenario. The P-value for the hypothesis test is then computed from the area under the null variance distribution function to the right of the measured value, σ_k.

Monte Carlo simulations for income-based gender inequality indicators

The income-based gender inequality indicator is based on average yearly income, and not on proportions. To account for this difference, the Monte Carlo procedure is modified, as follows. As before, k_{null} is estimated from the median of the measured gender inequality scores across all DAs. The average income of men and women used to calculate gender inequality scores is assumed to be normally distributed:

$$X_{im} \sim \mathcal{N}\left(X_{im\_null}, \sigma_{im\_null}\right)$$

$$X_{if} \sim \mathcal{N}\left(X_{if\_null}, \sigma_{if\_null}\right),$$

where X_{im} and X_{if} are the simulated income for men and women, respectively, in the ith DA. The parameters σ_{im_null} and σ_{if_null} are the standard errors of the average income obtained from census data, while X_{im_null} and X_{if_null} are the ‘true’ average income values under the null scenario for each DA, calculated from the gender inequality score (k_{null}) under the

condition that the total income of men and women equals the observed total income in each DA ($n_{im}X_{im} + n_{if}X_{if} = n_{im}X_{im\_obs} + n_{if}X_{if\_obs}$). Computation of P-values for the hypothesis test of income-based gender inequality indicators proceeds in the same manner as outlined for proportion-based indicators above.
